# Intracellular Signals Activated by Canonical Wnt Ligands Independent of GSK3 Inhibition and β-Catenin Stabilization

**DOI:** 10.3390/cells8101148

**Published:** 2019-09-25

**Authors:** Antonio García de Herreros, Mireia Duñach

**Affiliations:** 1 Programa de Recerca en Càncer, Institut Hospital del Mar d’Investigacions Mèdiques (IMIM), Unidad Asociada CSIC, and Departament de Ciències Experimentals i de la Salut, Universitat Pompeu Fabra, E-08003 Barcelona, Spain; 2 Departament de Bioquímica i Biologia Molecular, CEB, Facultat de Medicina, Universitat Autònoma de Barcelona, E-08193 Bellaterra, Spain

**Keywords:** Wnt signaling, Frizzled, LRP5/6, ROR2, β-catenin, STAT3, YAP/TAZ

## Abstract

In contrast to non-canonical ligands, canonical Wnts promote the stabilization of β-catenin, which is a prerequisite for formation of the TCF4/β-catenin transcriptional complex and activation of its target genes. This pathway is initiated by binding of Wnt ligands to the Frizzled/LRP5/6 receptor complex, and it increases the half-life of β-catenin by precluding the phosphorylation of β-catenin by GSK3 and its binding to the βTrCP1 ubiquitin ligase. Other intercellular signals are also activated by Wnt ligands that do not inhibit GSK3 and increase β-catenin protein but that either facilitate β-catenin transcriptional activity or stimulate other transcriptional factors that cooperate with it. In this review, we describe the layers of complexity of these signals and discuss their crosstalk with β-catenin in activation of transcriptional targets.

## 1. Introduction: Wnt Signaling Promotes β-catenin Stabilization

The relevance of the Wnt/β-catenin pathway (which we also will refer to as the “canonical” Wnt pathway) has been well established in embryonic development and in diseases such as cancer [1]. In a Wnt “off” state, cytosolic β-catenin (thus, not associated to E-cadherin and α-catenin in the adherens junctions) is bound to a destruction complex formed by the scaffolding proteins APC and Axin2 and the associated protein kinases GSK3 and CK1α (see Table 1 for a summary of proteins mentioned in this article) [1,2]. As a consequence, β-catenin is phosphorylated by GSK3 at Ser33 and Ser37, associates with βTrCP1 E3 ubiquitin ligase and is targeted for degradation by the proteasome. Canonical Wnt ligands initiate a series of reactions that lead to β-catenin stabilization, translocation of β-catenin to the nucleus where it interacts with TCF4 and other transcriptional factors, such as FOXO3 or LRH-1 [3,4,5] and transcription of genes bound by these complexes [2]. However, not all Wnt ligands signal through this pathway. A subset of these so-called non-canonical Wnts stimulates different molecular cascades that do not increase β-catenin protein levels; indeed, some of these pathways promote β-catenin downregulation [6]. The best known non-canonical pathway is the planar cell polarity pathway, which promotes the orientation of cells in vertebrate tissues. Both canonical and non-canonical Wnts use common receptors of the Frizzled (Fz) family but different co-receptors: LRP5 and LRP6 proteins, for the canonical ligands, and ROR1, ROR2, and RYK, for the non-canonical ligands. The goal of this review is to describe reactions triggered by canonical Wnts that are related to β-catenin transcriptional activity but not to β-catenin stability, and to compare them with those also promoted by non-canonical Wnts.

**Figure 1:**
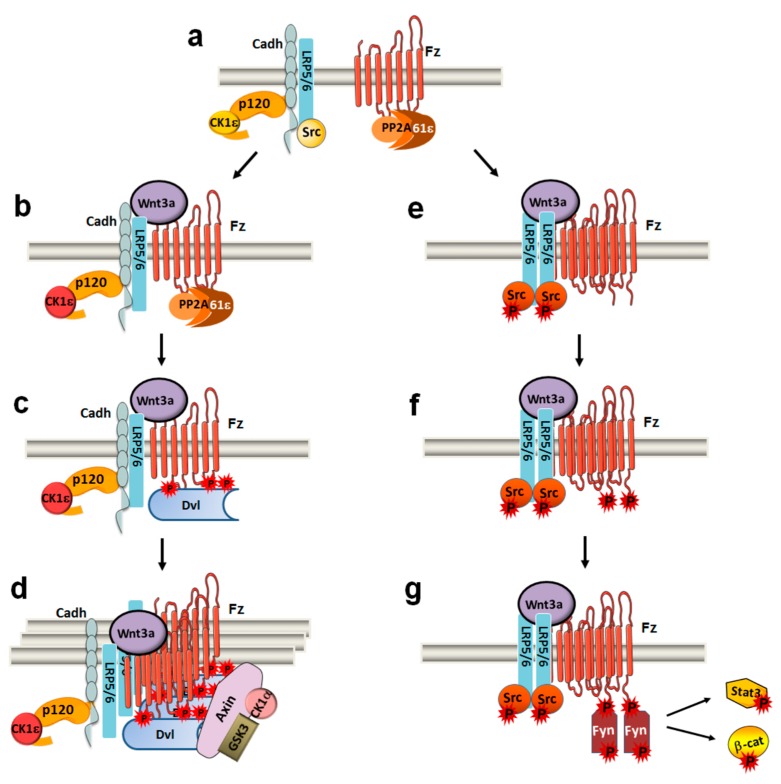
Canonical Wnt ligands induce two mutually exclusive Dvl2- and Fyn-dependent arms. (**a**) In Wnt OFF, LRP5/6 co-receptor interacts through p120-catenin and E-cadherin with inactive CK1ε and Src (both inactive kinases are shown in yellow). (**b**) Wnt3a promotes that PP2A phosphatase, associated to Fz2 through the PR61ε regulatory subunit, moves closer to CK1ε, and dephosphorylates and activates CK1ε (in orange). (**c**) CK1ε increases Dvl2 phosphorylation and its binding to Fz2. (**d**) Dvl2′s association leads to signalosome assembly, axin recruitment, and further responses of this pathway. (**e**) LRP5/6 dimerization promotes Src activation and (**f**) Src-dependent phosphorylation of Tyr552 in Fz2. (**g**) Phospho-Tyr552 binds and activates Fyn, promoting the phosphorylation of Stat3. Fyn also phosphorylates β-catenin Tyr142, releasing β-catenin from α-catenin and cadherin and thereby facilitating its transcriptional activity.

Receptor signaling is initiated by binding of the specific ligand and leads to the formation of protein aggregates known as signalosomes, which incorporate or release different factors along the progression of the pathway. Canonical Wnt signaling is initiated by the binding of factors such as Wnt3a or Wnt1 to a receptor complex comprising Fz and LRP5/6. As a first reaction, Wnt-induced assembly of the complex activates CK1ε by promoting the interaction of CK1ε, which is indirectly bound to LRP5/6 through E-cadherin/p120-catenin, with the Fz-associated PR61ε, a subunit of PP2A phosphatase that controls its activity (Figure 1a) [7,8]. This promotes dephosphorylation of the CK1ε C-terminal tail and activation of this protein kinase (Figure 1b). Wnt also promotes CK1ε binding to the RNA helicase DDX3, an interaction that stimulates CK1ε activity [9,10]. Curiously, CK1ε is also activated by non-canonical Wnt ligands [11,12]; in this case, CK1ε associates to the complex through its direct interaction with the non-canonical Wnt co-receptor ROR2 [13]. The role of DDX3 in the non-canonical activation of CK1ε has not yet been studied.

As a consequence of CK1ε activation, Dishevelled 2 (Dvl2) is recruited to the receptor complex by interacting with Fz (Figure 1c) [14,15]. As both canonical and non-canonical Wnts activate CK1ε, Dvl2 is recruited to Fz by both types of ligands. Besides interacting with other factors, Dvl2 polymerizes through its DIX domain [16]. Thus, binding of Dvl2 to the receptor/co-receptor complex allows assembly of the signalosome (Figure 1d). This process is enhanced by proteins such as TMEM59, which interacts with Fz and LRP5/6 [17]. Dvl2 also binds and activates phosphatidylinositol 4-kinase IIα and phosphatidylinositol 4-phosphate 5-kinase 1β, generating PtdIns(4,5)P_2_ [18]. PtdIns(4,5)P_2_ favors receptor aggregation through the AP2 clathrin adaptor complex [19] and also promotes AMER/WTX–mediated association of CK1γ with LRP5/6 [20]. CK1γ is also required for the non-canonical PCP pathway [21]. This is the diverging point of the two signaling pathways, as CK1γ phosphorylates LRP5/6 in Thr1479, creating a docking site for axin and enhancing the interactions of axin and the associated protein kinases CK1α and GSK3 with the LRP5/6 complex (Figure 1d) [22,23]. The precise substrates of CK1γ in the non-canonical PCP pathway have not yet been characterized. 

Axin association with LRP5/6 causes GSK3 inhibition and precludes β-catenin phosphorylation at Ser37, β-catenin binding to βTRCP1 ubiquitin ligase and β-catenin targeting to the proteasome. However, the mechanism behind the GSK3 inhibition is still a matter of discussion, and several have been proposed [24,25]. According to one model, Wnt signaling promotes axin dephosphorylation by the PP1 phosphatase and reduces axin binding to β-catenin and GSK3, thereby preventing the action of this kinase on β-catenin [26,27,28]. Another proposal is that axin oscillates between two states: either an open state that is permissive for β-catenin binding, or a closed state that prevents β-catenin association and degradation [29]. The switch between these two states would depend on axin’s interaction with the signalosome (and specifically, with Dvl2), which decreases its capability to polymerize [30], as well as on axin’s phosphorylation state, which is a consequence of the combined actions of GSK3, CK1α and PP1 phosphatase. A third mechanism proposes a direct inhibition of GSK3 by LRP5/6: axin-bound GSK3 catalyzes the LRP5/6 phosphorylation of PPPSPxS motifs (such as Ser1490) to create an inhibitory site for GSK3 [31,32,33,34]. This site would then compete for interactions with other substrates, such as β-catenin. Another model suggests that the important step is not inhibition of β-catenin Ser37 phosphorylation by Wnt signaling but rather the Wnt signaling–induced degradation of phosphorylated-β-catenin [35]. According to this model, access of βTrCP1 to phosphorylated β-catenin is hindered upon Wnt stimulation. 

A distinct type of mechanism based on the endocytic sequestration of GSK3 has also been proposed [36]. This model is supported by previous results indicating that caveolin-dependent LRP5/6 internalization is required for β-catenin accumulation [37,38,39]. According to this model, the Wnt signalosome, comprising LRP5/6, Dvl2, axin and GSK3, is endocytosed into acidic vesicles, called multivesicular bodies (MVB) [36].Therefore, GSK3’s access to its cellular substrates (including β-catenin) would be prevented. Internalization of LRP5/6 in MVB requires the release of N or E-cadherin from the signalosome; notably, inhibition of this internalization affects β-catenin stabilization but does not totally prevent it, leading to speculation that several of the mechanisms mentioned above may co-exist [40]. 

If axin-bound GSK3 indeed constitutes a relevant part of the total kinase present in the cell (which still needs to be verified), GSK3 inhibition would be expected to modulate the half-life of many of its substrates, and not only β-catenin. Indeed, Wnt increases the stability of many proteins [36,41]. This process of extensive stabilization of proteins by Wnt has been named Wnt/STOP [42] and is an example of a Wnt effect that is independent of β-catenin stabilization. Moreover, as GSK3 is a pleiotropic protein kinase, its inhibition produces many other effects that are not necessarily due to degradation of its substrates. For instance, GSK3 phosphorylates TSC2, thereby promoting its binding to, and inhibition of, the mTOR complex 1 (TORC1); in agreement with this, Wnt-induced GSK3 inhibition blocks TSC2 phosphorylation and activates TORC1 and protein translation [43]. Although these Wnt effects are independent of β-catenin, they are due to GSK3 inhibition and will not be further addressed in this review. 

## 2. STAT3 Transcriptional Activity is Stimulated by Canonical and Non-Canonical Wnts 

Even though canonical and non-canonical Wnts have opposite effects on the level of β-catenin (by up- or downregulating it, respectively) [6], both canonical and non-canonical Wnt ligands stimulate migration and invasion in several cellular systems. This apparent conundrum can likely be resolved by attributing these effects to other signals commonly activated by Wnt factors, such as RAC1/JNK2 kinase (see below). Moreover, similar to non-canonical ligands [44], canonical Wnts such as Wnt3a rapidly stimulate Fyn activation, STAT3 phosphorylation and the transcription of genes involved in the epithelial-to-mesenchymal transition and acquisition of invasiveness (Figure 1g) [45]. This effect is inhibited by DKK1 and mimicked by ectopic expression of LRP5/6, indicating that it happens through the receptor of canonical Wnts [45]. Mechanistically, Fyn activation is rapid, precedes β-catenin accumulation and is dependent on Fyn binding to the Fz-receptor phosphorylated at Tyr552 in its C-terminal tail [44] (Figure 1g). Src is the Tyr protein kinase responsible for phosphorylation of this residue, which is present only in three Fz receptors (Fz1, -2 and -5) (Figure 1f) [45]. Consequently, Src kinase activity is activated both by canonical and non-canonical ligands (Figure 1e) [45,46,47]. Src co-immunoprecipitates with the co-receptors of both canonical (LRP5/6) and non-canonical (ROR2) Wnts [45,48,49], suggesting that it also participates in both signalosomes, although its interaction with ROR2 seems to be more direct than with LRP5/6. It is remarkable that activation of this Src/Fyn/STAT3 pathway by Wnt is independent of CK1ε activity and Dvl2 [45]. This indicates that extensive polymerization of the receptor complex is not required for Src activation. However, binding of Wnt ligands promotes dimerization of the receptor complex, as Wnt interacts with Fz with 1:2 stoichiometry [50,51]; this receptor dimerization might promote the intermolecular phosphorylation and activation of Src protein (Figure 1e). Accordingly, ectopic overexpression of either LRP6 or ROR2 promotes Src activation and STAT3 phosphorylation [45]. In any case, we cannot discard that a Fz-bound factor is also relevant for Src activation. 

The interaction between Fz2 and Fyn does not require Dvl2 and is incompatible with the association between Fz2 and Dvl2, further demonstrating that these two interactions (Fz2-Fyn and Fz2-Dvl2) define two distinct, mutually antagonistic arms of Wnt signaling [45] (Figure 1). Previous results have suggested that Src tyrosine kinases interfere with canonical Wnt signaling. For instance, Src activation blocks β-catenin–dependent transcription caused by LRP5/6 ectopic expression or by Wnt3a [49]; while this might be due to the direct phosphorylation of LRP5/6 by Src, it could also be a consequence of an enhanced Fz phosphorylation at Tyr552 blocking the Dvl2-dependent molecular cascade. 

Although Fyn controls the transcription of many canonical Wnt target genes, full activation of the Wnt3a pathway depends not only on activation of Dvl2 but also of Src/Fyn/STAT3–dependent branch [45]. This likely is due to an additional action of Fyn: the phosphorylation of Tyr142 in β-catenin disrupts its interaction with α-catenin and helps to mobilize β-catenin [52]. This PTyr142–β-catenin has a stronger affinity for Pygo/BCL9 and contributes to its transcriptional activation [53,54]. This mobilization of β-catenin might be relevant for genes whose transcription require a high number of β-catenin/TCF4 complexes or β-catenin bound to other cofactors. In contrast, other genes might only need the action of newly synthetized β-catenin. 

Moreover, through STAT3 phosphorylation, Fyn activates genes containing STAT3-responsive elements in their promoters. A requirement for STAT3 in the transcription of β-catenin targets has been described [55]. However, by mobilizing STAT3, canonical Wnt ligands can activate genes that do not contain β-catenin/TCF binding sites in their promoters, such as *SNAI1* [45]. 

Activation of Fyn and STAT3 phosphorylation requires phosphorylation of Fz at Tyr552, which is only present in the Fz receptor members Fz1, -2 and -7 [45]. Thus, this pathway is exclusively activated by Wnt in cells that express these receptors, introducing the concept of Fz receptor selectivity, which not only refers to its binding to different ligands as previously shown [56], but also to the specific activation of signaling pathways. In this respect, modulation of other responses of Wnt, such as YAP1/TAZ activity, is differentially sensitive to the presence of Fz1, -2 or -5 in the receptor complex [57]. 

## 3. JNK2 and PAK Dependent Activation of β-Catenin Nuclear Transport

**Figure 2:**
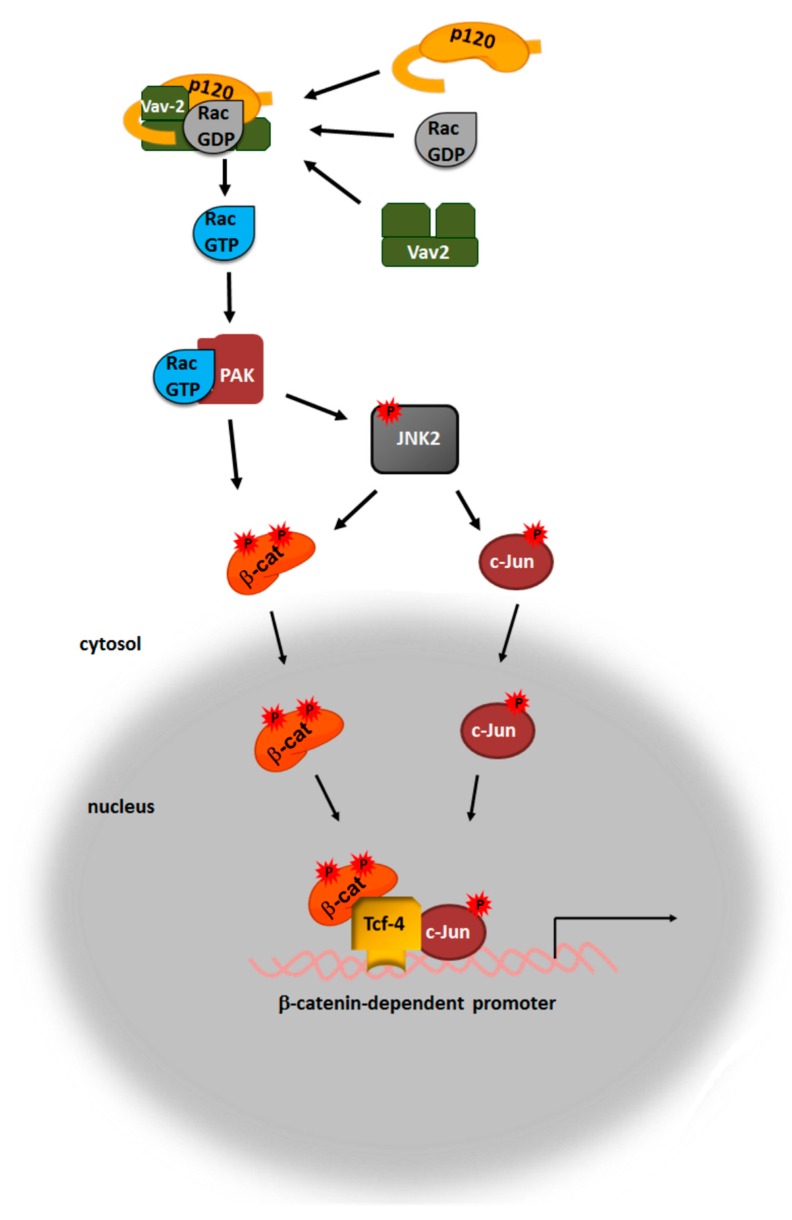
Rac1 activation by p120-catenin/Vav2. p120-catenin released from the association with E-cadherin binds to Rac1-GDP and Vav2. The p120-catenin interaction facilitates Rac1 activation by increasing the local concentration of this protein in the vicinity of Vav2 as well as by eliminating the restriction caused on Vav2 activity by the acidic and calponin-homology domains. Rac1-GTP activates the PAK kinase, which upregulates the activity of the Ser/Thr JNK2 kinase. Both PAK and JNK2 phosphorylate β-catenin, thereby favoring either its translocation to the nucleus and/or its nuclear retention. Moreover, JNK2 also phosphorylates c-Jun and promotes its nuclear import, thereby facilitating its interaction with the β-catenin/Tcf4 complex and the transcriptional activation.

Besides requiring protein stabilization, transcriptional activity of β-catenin involves its transport to the nucleus. This traffic requires the activity of the small GTPase Rac1, which is up-regulated by canonical Wnt ligands [58,59,60]. When activated and bound to GTP, Rac1 stimulates PAK protein kinase, which subsequently phosphorylates and activates JNK2 (Figure 2) [59]. Rac1 and JNK2 are also stimulated by non-canonical Wnt ligands [61]. It has been proposed that in canonical Wnt, JNK2 promotes β-catenin translocation to the nucleus by phosphorylating Ser191 and Ser605 [59]. Additionally, PAK1 also directly phosphorylates β-catenin at Ser675, contributing to its stabilization (Figure 2) [62]. Some have questioned whether phosphorylation of Ser191 and Ser605 in β-catenin has an impact on β-catenin nuclear translocation, suggesting that instead it stimulates β-catenin presence in the nucleus by enhancing its interaction with LEF1 as well as potentially with other transcription factors [63]. Alternatively, or additionally, β-catenin traffic to the nucleus might be boosted by its phosphorylation at Tyr654 and Tyr142, two modifications that decrease its affinity for E-cadherin and α-catenin, respectively [52,64].

Several mechanisms of Rac1 activation by Wnt have been proposed. One of the first mechanisms suggested that Dvl2 and phosphatidylinositol 3-kinase are required for Rac1 activity, although no precise mode of activation was described [59]. Indeed, reports have now shown that Dvl2 interacts with Rac1 [58,65] as well as with the Rac1 guanine nucleotide exchange factor TIAM1 [65]. TIAM1 is necessary for the activation of Rac1 by the non-canonical Wnt5a and likely also for the interaction of Rac1 with Dvl2. Wnt5a enhances the TIAM1-Dvl2 interaction; surprisingly, this increased binding is associated to Dvl2 dephosphorylation, a modification opposite to that detected upon Wnt activation [65]. Another Dvl2-associated protein, Daple, is also involved in Rac1 activation by non-canonical Wnt5a, in this case by enhancing the interaction of Dvl2 with PKCλ/ι [66]. 

An alternative mechanism for Rac1 activation by canonical Wnts depends on Rac1’s interaction with p120-catenin that is discharged from the signalosome [67]. Upon Wnt stimulation, p120-catenin is phosphorylated by axin-bound CK1α at Ser268 and Ser269, which disrupts its interaction with cadherin and releases it to the cytosol [68,69]. Cytosolic p120-catenin binds to Vav2 and Rac1 and promotes Rac1 activation (Figure 2) [67,70,71]. Besides increasing the local concentration of Rac1 and favoring Vav2/Rac1 binding, it is possible that interaction with p120-catenin releases the Vav2 catalytic Dbl homology domain from the coordinated inhibition by the acidic and calponin homology domains [72], mimicking the effect of Vav2 phosphorylation in the acidic domain. This mode of Rac1 activation by p120-catenin might be also shared by other members of the family, such as the ARVCF (armadillo repeat gene deleted in velo-cardial-facial syndrome) protein, as this closely related protein can rescue the effects of p120-catenin depletion in *Xenopus laevis* development [73]. Moreover, depletion of both proteins can be compensated by a dominant-active form of Rac1 [73]. The possible interaction of TIAM1 with p120-catenin also deserves to be studied. 

Finally, the Rac1/PAK/JNK2 pathway has been implicated in β-catenin phosphorylation and trafficking to the nucleus; however, this is not the only activity of this molecular axis in Wnt signaling. Another substrate of JNK2, c-Jun, interacts with TCF4 and cooperates with β-catenin to regulate gene transcription (Figure 2) [74]. Accordingly, c-Jun is required for a full response to canonical ligands in cells and zebrafish embryos [75], suggesting that activation of Rac1 might control gene transcription not only by promoting β-catenin nuclear localization. Curiously, the interaction of c-Jun with β-catenin/TCF4 in the nucleus is mediated by Dvl2, which is also detected to be associated to gene promoters [75]. The basis of this nuclear localization of Dvl2, and its possible regulation by Wnt, has not yet been characterized; it has only been reported that it is promoted by Forkhead box transcriptional factors FOXK1 and FOXK2 and by IQGAP1, all of which are proteins that directly interact with Dvl2 [76,77]. Besides activating β-catenin/TCF4/c-Jun dependent transcription, nuclear Dvl2 also binds p65 and represses NFκB function [78]. 

## 4. Canonical Wnts Prevent the Action of the Kaiso Inhibitor

**Figure 3:**
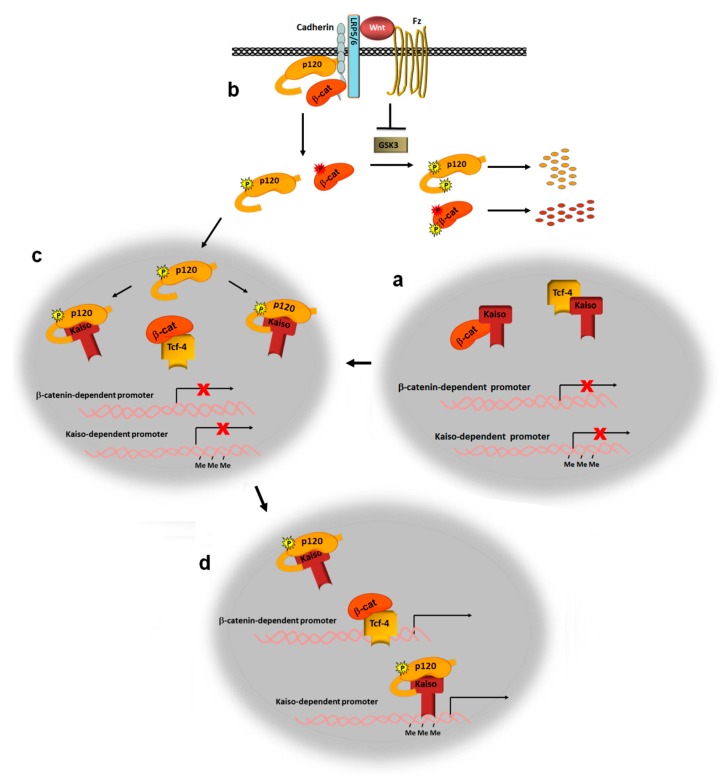
Wnt controls kaiso transcriptional repressor through p120-catenin. In the Wnt “off” state (**a**), kaiso represses β-catenin/TCF4 transcription through its interactions with TCF4, which precludes its binding to DNA, as well as with β-catenin, which inhibits its interaction with TCF4. Upon Wnt stimulation (**b**), p120-catenin and β-catenin that are released from the signalosome through their phosphorylation at Ser268/269 and Tyr142, respectively, are stabilized by the Wnt-induced inhibition of GSK3; this then prevents p120-catenin and β-catenin degradation. In the nucleus (**c**), p120-catenin binds kaiso and disrupts TCF4/kaiso and β-catenin/kaiso interactions, allowing the β-catenin/TCF4 complex to bind its target promoters (**d**). Note that kaiso binding to methylated CpG sequences is not prevented by p120-catenin.

The transcriptional factor kaiso inhibits the expression of many canonical Wnt target genes. This was initially attributed to its direct interaction with 5′-CTGCNA-3′ motifs present in Wnt target promoters, such as *Matrilysin* [79,80,81]. However, other authors have demonstrated that kaiso binds to these motifs but with low affinity [82], and they have proposed alternative mechanisms of inhibition. For instance, inhibition of the Wnt pathway by kaiso may be dependent on its association with TCF3/4, which precludes the binding of this factor to DNA (Figure 3a) [82]. Kaiso also interacts with β-catenin, thus preventing its interaction with TCF4 (Figure 3a) [83]. Finally, kaiso also represses the expression of other genes that are not transcriptionally dependent on β-catenin, through kaiso’s interaction with methylated CpG sequences in their promoters [84,85,86]. Therefore, in non-activated cells, kaiso is present in the nucleus, where it interacts with TCF4 and precludes its binding to DNA, as well as with any residual β-catenin that might have escaped from APC-mediated degradation. Thus, kaiso prevents β-catenin from associating with TCF4, as well as the binding of TCF4 to DNA. 

Upon canonical Wnt stimulation, the kaiso inhibition is eliminated mainly through the action of p120-catenin that becomes phosphorylated, is released from cadherin and translocates to the nucleus through the action of nuclear localization sequences present in the Armadillo domain (Figure 3b) [87,88]. Besides disrupting p120-catenin interaction with E-cadherin, Wnt-induced Ser268 and Ser269 phosphorylation enhances p120-catenin binding to kaiso [83]. The interaction with p120-catenin precludes the association of kaiso with TCF4 and β-catenin and permits the formation of the β-catenin-TCF4 complex and its binding to DNA (Figure 3c). Remarkably, p120-catenin does not alter kaiso interaction with methylated DNA sequences [83] probably as consequence of the different kaiso amino acid sequences involved in binding to DNA and Tcf-4 [82,84]. Therefore, disruption of TCF4/kaiso complex by p120-catenin, besides enabling TCF4 to activate its transcriptional targets also facilitates kaiso interaction with promoters containing CpG islands, such as *CDKN2A* (Figure 3d). Adding a further level of complexity, kaiso can be converted from a repressor to an activator by sumoylation [89]. 

It should also be taken into consideration that, in the nucleus, p120-catenin not only associates with kaiso but also with other transcription factors, such as Glis2 [90] and REST/CoREST [91]. p120-catenin binding to REST/coREST prevents this complex from binding its target promoters and thus from repressing the corresponding genes. Therefore, canonical Wnt signaling might also regulate the repressive action of REST/coREST. 

This nuclear function of p120-catenin, and likely also its action on Rac1, is controlled by canonical Wnt ligands that not only promote its release from cadherin but also enhance the stability of the cytosolic p120-catenin protein. This effect is dependent on frodo, a Dvl2-associated protein that also interact with p120-catenin [92]. Wnt controls p120-catenin stability also by inhibiting its phosphorylation in its N-terminal domain by CK1α and GSK3, in a manner analogous to that of β-catenin [93] (Figure 3b). Thus, these two catenins exhibit several similarities: both are present in the cell junctions and, once released to the cytosol, they need to be stabilized in order to translocate and function in the nucleus. Moreover, p120-catenin stability is also enhanced by DYRK1-mediated phosphorylation, further facilitating binding to kaiso and preventing kaiso’s repression of its target genes [94]. 

## 5. Wnt Ligands Modulate YAP/TAZ Transcriptional Activity

**Figure 4:**
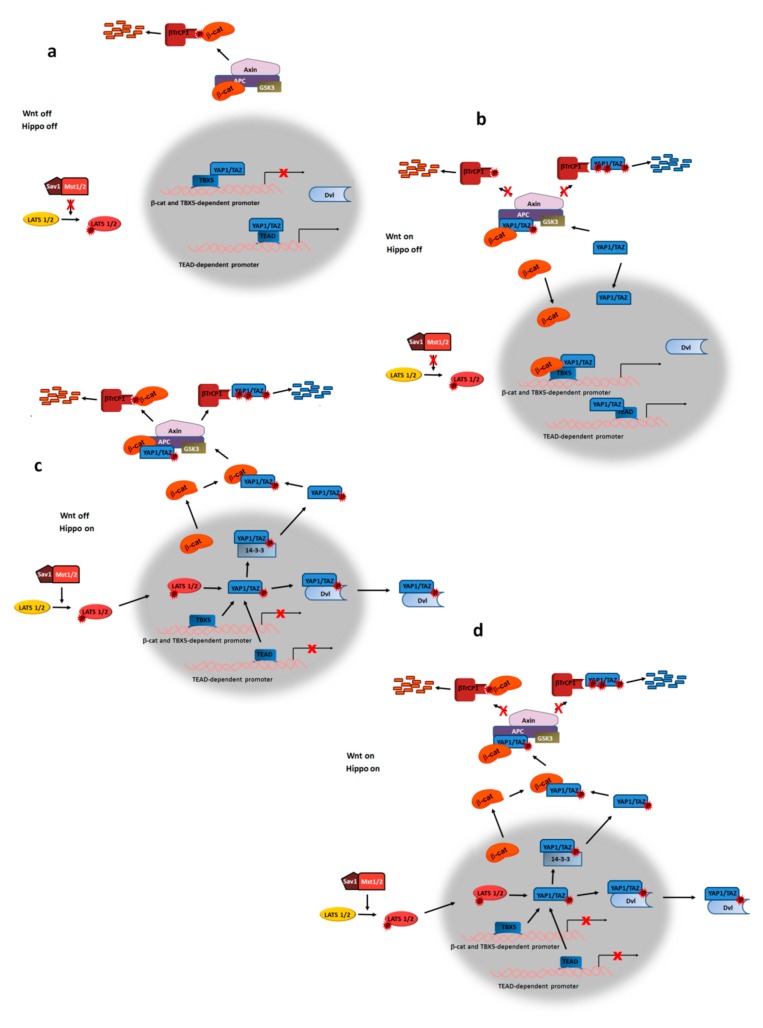
Antagonism between Wnt and Hippo signaling pathways. (**a**) In cells with inactive Hippo signaling (and therefore inactive LATS1/2 protein kinase), Hippo YAP/TAZ is present in the nucleus and interacts with the TEAD transcriptional factor, thereby inducing transcription of sensitive promoters. β-catenin is degraded by the proteasome after GSK3 phosphorylation, dependent on β-catenin binding to APC/axin complex. Inactive protein kinases are depicted in yellow, and active ones, in orange. (**b**) Wnt activation stabilizes β-catenin and promotes its translocation to the nucleus. Once in the nucleus, β-catenin associates not only with TCF4 and other transcriptional factors but also to a complex formed by TBX5 and YAP1/TAZ, thereby promoting the transcription of proliferation-related genes. (**c**) When Hippo is induced, active Mst1 kinase bound to the Sav1 scaffolding protein phosphorylates and activates the protein kinase LATS1/2. This protein kinase then modifies YAP1/TAZ, facilitating its export from the nucleus through the action of the 14-3-3 chaperone and likely also Dvl2. Cytosolic YAP1/TAZ also interacts with β-catenin, precluding its transport to the nucleus. In the cytosol, YAP1/TAZ is further phosphorylated by GSK3, creating a phosphodegron for the βTrCP1 ubiquitin ligase and targeting YAP1/TAZ for proteasomal degradation. Interactions with β-catenin, axin, and APC are also required for YAP/TAZ phosphorylation by GSK3. (**d**) Wnt activation precludes β-catenin and YAP/TAZ phosphorylation and degradation, but it does not prevent YAP export from the nucleus and does not (at least not more than minimally) activate TEAD- or TBX5-sensitive promoters.

The canonical Wnt pathway also presents a high degree of crosstalk with Hippo signaling that controls gene transcription through the actions of YAP1 and TAZ. When Hippo is not active, YAP1 and TAZ are present in the nucleus and associate with DNA through the TEAD transcription factor, promoting the expression of proliferative and survival genes (Figure 4a) [95,96]. In the nucleus, YAP1 also interacts with β-catenin when Wnt signaling is “on” and forms a complex with the TBX5 transcription factor that is essential for expression of genes related to colon tumorigenesis (Figure 4b) [97]. YAP also controls the transcription of several members of the JAK2/STAT3 pathway including several cytokines; accordingly, repression of YAP by the Hippo kinases Mst1/2 decreases STAT3 phosphorylation [98,99]. The activity of the YAP1/TBX5/β-catenin complex is controlled through YAP1 phosphorylation by the tyrosine kinase Yes; this phosphorylation facilitates its localization to the promoters of its target genes. Moreover, methylation of YAP1 at Lys494 enhances β-catenin’s binding and nuclear localization [100]. 

Activation of the Hippo pathway represses the transcriptional activity of YAP1 and TAZ, promoting its export from the nucleus. Stimulation of the pathway by growth inhibitory signals activates the Sav1/Mst1/2 protein kinase complex, which then promotes the phosphorylation and activation of another protein kinase, LATS1/2 (Figure 4c). LATS1/2-dependent phosphorylation of YAP and TAZ causes their nuclear export mediated by their binding to 14-3-3 proteins; in the cytosol, YAP1 and TAZ are further phosphorylated by GSK3, inducing their binding to βTrCP1 and their degradation [96]. LATS1/2 activity on YAP1 and TAZ is inhibited by Rac1 or RhoA, increasing the transcriptional activity of the YAP1/TAZ/TEAD complex [57]. This upregulation in YAP1 activity mediated by Rac1, and the above-mentioned stimulation of this GTPase by Wnt, suggest that canonical ligands mightenhance YAP1-dependent transcription.

Other results also provide evidence for the antagonism between Wnt signaling, which causes β-catenin-dependent transcription, and Hippo activation, which inhibits the YAP1/TAZ/TEAD complex. For instance, phosphorylated YAP1 interacts with β-catenin in the cytosol, preventing its translocation to the nucleus and activation of its target genes (Figure 4c,d) [101]. The member of the β-catenin–destruction complex APC participates in Hippo signaling, acting as a scaffold protein for Sav1 and LATS1/2 [102]; consequently, APC mutations activate Wnt signaling bypreventing β-catenin degradation and also inhibit Hippo as they preclude YAP1 phosphorylation. Moreover, degradation of the β-catenin and YAP/TAZ proteins is also interdependent. The TAZ protein is degraded through the same destruction complex that acts on β-catenin; in the absence of Wnt, TAZ is maintained at low levels by the coordinated action of APC, Axin, GSK3 and phosphorylated β-catenin, which facilitates βTrCP1’s interaction with TAZ and proteasomal degradation [103]. Wnt signaling prevents β-catenin’s phosphorylation and degradation and also impairs TAZ’s destruction (Figure 4d). Some authors have proposed that the effect is reciprocal, and that the presence of YAP/TAZ in the complex also enables the recruitment of βTrCP1 and β-catenin inactivation [104]. 

YAP1 also interacts physically and functionally with Dvl proteins (Figure 4). Some authors have proposed that the YAP1-Dvl association restricts Dvl activity by preventing its nuclear translocation [105]. As described above, nuclear Dvl2 facilitates β-catenin/TCF4 interaction with c-Jun, an association required for the expression of its canonical target genes [75]. Conversely, Yook and others have reported that Dvl is responsible for the nuclear export of phosphorylated YAP1 (Figure 4c and d); inhibition of Dvl nuclear export leads to an increase in nuclear YAP as well as in TEAD transcriptional activity [106]. 

YAP and TAZ are also controlled by the non-canonical or alternative Wnt pathway. Acting through the Fz/ROR complex, Wnt ligands prevented YAP1 phosphorylation through a signaling pathway involving Gα_12/13_ and Rho that promote the inhibition of LATS1/2 [57]. Both Gα_12/13_ and Rho are known inhibitors of LATS1/2 [107,108]; however, their involvement in Wnt signaling remains controversial [109]. Wnt5a also interferes with Hippo signaling at another level, as it induces transcription of *SIAH2*, a gene encoding a ubiquitin ligase that targets LATS2 and prevents YAP1 phosphorylation [110]. Interestingly, as a consequence of LATS1/2 inhibition and YAP1 activation, non-canonical Wnt5a increases the synthesis of DKK1 and other inhibitors of canonical β-catenin-dependent Wnt signaling and thereby prevents the effects of canonical Wnts [57]. 

These results also demonstrate a general antagonism between the actions of Wnt ligands triggering the canonical and non-canonical pathways; for instance, through the stimulation of SIAH2, that besides LATS1/ also targets b-catenin for degradation [6], non-canonical Wnt5a down-regulates β-catenin and represses β-catenin–dependent transcription [6]. However, the effects of SIAH2 expression are more complex: it also promotes YAP1 transcriptional activity, which cooperates with β-catenin in the nucleus [97] and even enhances β-catenin signaling through the degradation of axin when this protein is not bound to the destruction complex [111]. It is possible that the action of SIAH2 on the different substrates is mediated post-translationally. Nonetheless, the cooperation or antagonism between canonical and non-canonical Wnts likely depends on the different pathways triggered by these factors, for instance in the extent of activation of common (STAT3 activation) versus specific responses. 

## 6. Concluding Remarks

Although the best studied response to canonical Wnts is β-catenin stabilization, the signaling pathways triggered by these factors are not limited to this effect. A full transcriptional response requires other reactions necessary for a proper β-catenin translocation to the nucleus (such as the activation of Rac1 and JNK2), for the inactivation of negative cofactors (such as kaiso), and for the stimulation of additional transcriptional factors that cooperate with the β-catenin/TCF4 complex (such as STAT3). Moreover, Wnt signaling impacts the stability of other proteins, such as TAZ, indicating the pleomorphic effect of this pathway. Finally, these effects of canonical Wnt ligands are in some cases shared by other Wnts that stimulate the non-canonical pathway: for instance, both types of ligands stimulate Rac1 and STAT3. In any case, many different aspects of this transduction cascade remain to be clarified to better explain the crosstalk with other pathways and to fully characterize the molecular reactions underlying them.

## Figures and Tables

**Table 1:** Proteins involved in Wnt or Hippo signaling.

Name	Function
**14-3-3**	Chaperone proteins binding YAP/TAZ after phosphorylation by LATS1/2 and promoting their nuclear export
**α-catenin**	Linking protein between β-catenin-E-cadherin complex that regulates actin filament assembly.
**AMER/WTX** (APC Membrane Recruitment Protein 1) (Wilms Tumor On The X)	Protein acting in Wnt pathway that facilitates the formation of complex composed by Axin, GSK3 and LRP.
**AP2** (Adaptor protein 2)	Adaptor protein which contributes to clathrin-mediated endocytosis.
**APC** (Adematous polyposis coli)	Negative regulator of the Wnt pathway that controls β-catenin degradation. Also regulates the Hippo pathway controlling TAZ degradation.
**ARVCF** (Armadillo Repeat Protein Deleted In Velo-Cardio-Facial Syndrome)	Member of the armadillo protein family related to p120-catenin and involved in Rac1 activation
**Axin**	Member of the β-catenin and TAZ destruction complex. Negative regulator of the canonical Wnt pathway.
**BCL9**	β-catenin cofactor relevant for the transcription of canonical Wnt target genes
**β-catenin**	Protein involved in the regulation of cellular adhesion and gene transcription. Acts as an intracellular signal transducer in the Wnt signaling cascade.
**βTrCP1** (Beta-Transducin Repeat Containing E3 Ubiquitin Protein Ligase)	Ubiquitin ligase that regulates the Wnt and Hippo pathways through β-catenin and TAZ degradation, respectively.
**Caveolin**	Membrane protein involved in Wnt-dependent LRP5/6/Dvl2/Axin/GSK3 internalization and required for β-catenin accumulation.
**CK1α** (Casein Kinase 1 alpha)	Protein kinase that associates to Axin and participates in the β-catenin degradation complex phosphorylating this protein and Axin
**CK1γ** (Casein Kinase 1 gamma)	Protein Kinase that is recruited to the Wnt signalosome and promotes LRP5/6 phosphorylation
**CK1ε** (Casein Kinase 1 epsilon)	Protein kinase rapidly activated canonical and non-canonical Wnt ligands and required for Dvl2 association to the receptor complex.
**Daple**	Protein involved in Rac1 activation by non-canonical Wnt
**DDX3** (DEAD-Box Helicase 3 X-Linked)	RNA helicase activated by Wnt signaling relevant for CK1ε activation
**DKK1** (Dickkopf WNT Signaling Pathway Inhibitor 1)	Extracellular protein inhibitor of the canonical Wnt pathway.
**Dvl2** (Dishevelled 2)	Cytosolic adaptor protein involved in canonical and non-canonical Wnt pathway. Wnt activation recruits Dvl to the membrane. It participates in assembling the signalosome. It is also detected in the nucleus bound to p65, FOXK and c-Jun
**DYRK1** (Dual Specificity Tyrosine Phosphorylation Regulated Kinase 1A)	Protein tyrosine kinase that enhances p120-catenin stability and relieves Kaiso repression
**E- and N-cadherin**	Transmembrane proteins directly bound to β-catenin, p120-catenin and LRP5/6 at the adherens junctions essential for intercellular interactions
**FOXK1/2** (Forkhead Box K1)	Transcriptional factor involved in the nuclear localization of Dvl2
**FOXO3** (Forkhead Box O3)	Transcriptional factor that upon binding to β-catenin behaves as a transcriptional activator
**Frodo**	Dvl2-associated protein that also interacts with p120-catenin
**Fyn**	Member of the Src family of protein kinases activated by canonical and non-canonical Wnts and required for Stat3 phosphorylation and transcriptional action
**Fz** (Frizzled)	Family of membrane receptors involved in canonical and non-canonical Wnt signaling.
**Gα_12/13_** (G Protein Subunits Alpha 12 and 13)	Family of heterotrimeric G proteins required for Rho activation and the inhibition of Lats1/2 in the Hippo pathway.
**Glis2**	Transcriptional repressor bound to p120-catenin in the nucleus
**GSK3** (Glycogen Synthase Kinase 3)	Serine/threonine protein kinase which phosphorylates β-catenin targeting it for degradation. Inhibited by canonical Wnt
**IQGAP1** (IQ Motif Containing GTPase Activating Protein 1)	Modulator of Dvl2 nuclear localization in Wnt signaling.
**JNK2** (c-Jun N-Terminal Kinase 2)	Protein kinase activated by canonical Wnt that modifies β-catenin and c-Jun facilitating gene transcription
**c-Jun**	Substrate of JNK2 that interacts with Tcf4/β-catenin to regulate gene transcription.
**Kaiso**	Transcriptional repressor of canonical Wnt target genes. Interacts with TCF4, β-catenin and p120-catenin.
**LATS1/2** (Large Tumor Suppressor Kinase 1 and 2)	Protein kinase that phosphorylates YAP1 and TAZ upon activation of the Hippo pathway promotes their nuclear export.
**LRH1** (Liver Receptor Homolog-1)	Nuclear orphan receptor that interacts with β-catenin independent of TCF4. Is activated by β-catenin; also promotes activation of β-catenin/TCF4 complex.
**LRP5/6** (LDL Receptor Related Protein 5 and 6)	Membrane co-receptor involved in canonical Wnt signaling
**Mst1/2** (Mammalian STE20-Like Protein Kinase 1 and 2)	Protein kinase stimulated by the Hippo pathway that activates Lats1/2.
**p120-catenin**	Protein of the armadillo family essential for canonical and non-canonical Wnt signaling. Interacts and stabilizes in the plasma membrane Cadherin/LRP and Ror2. Also binds and regulates CK1ε, Rac1 and Kaiso
**p65** (Rel A proto-oncogene)	Transcription factor member of the NF- B complex. Binds Dvl2 in the nucleus.
**PAK1** (P21 (RAC1) Activated Kinase 1)	Protein kinase stimulated by Rac1 in canonical and non-canonical Wnt. Activates JNK2 and promotes β-catenin translocation to the nucleus.
**PKC-λ** **(Protein Kinase C-λ)**	Atypical PKC that interacts with Dvl2
**PR61ε** (Protein Phosphatase 2, Regulatory Subunit B (B56), Epsilon Isoform)	Regulatory subunit of the PP2A phosphatase. Required for the activation of CK1ε at the initiation of the canonical and non-canonical Wnt signaling.
**Pygo** (Pygopus Family PHD Finger)	Transcriptional factor that binds to Bcl9 enhancing β-catenin transcriptional activation
**Rac1**	Small GTPase activated by canonical and non-canonical Wnts. Activates PAK. Inhibits LATS1/2 up-regulating YAP1/TAZ transcriptional activity
**REST/CoREST** (RE1 Silencing Transcription Factor)	Transcriptional complex modulated by p120-catenin
**RhoA**	Small GTPases that inhibits LATS1/2 activity in the Hippo pathway
**Ror1,2** (Retinoid-Related Orphan Receptor)	Tyrosine kinase-like orphan transmembrane co-receptor required for non-canonical Wnt signaling
**Ryk**	Transmembrane co-receptor involved in the non-canonical Wnt pathway.
**Sav1** (Salvador Family WW Domain Containing Protein 1)	Regulator of Mst1 in the Hipo pathway required for LATS1 phosphorylation and activation
**SIAH2** (Seven In Absentia (Drosophila) Homolog 2)	E3 ubiquitin-protein ligase stimulated by non-canonical Wnt involved in β-catenin degradation independent on GSK3
**Snail1**	Transcription factor activated by canonical and non-canonical Wnt that induces epithelial to mesenchymal transition.
**Src**	Non-receptor tyrosine protein kinase that binds to Wnt co-receptors LRP5/6 and Ror1 and is stimulated by canonical and non-canonical Wnts.
**STAT3** (Signal Transducer And Activator Of Transcription 3)	Transcription factor stimulated by canonical and non-canonical Wnts through the Fz/Fyn branch that activates genes involved in EMT and cell invasion.
**TAZ**	Transcriptional factor repressed by the Hippo pathway.
**TBX5** **(T-Box 5)**	Transcriptional factor that interacts with β-catenin and YAP1 in the nucleus and promotes transcription of genes related to colon tumorigenesis
**TCF4** (T-cell factor 4)	Transcription factor involved in canonical Wnt signaling which binds to DNA and recruits β-catenin.
**TEAD** (TEA Domain Transcription Factor)	Transcription factor. Forms a complex with YAP1 and TAZ in the nucleus and promotes the expression of proliferative genes.
**TIAM1** (T Cell Lymphoma Invasion And Metastasis 1)	Rac1 Guanosine exchange factor involved in Wnt-dependent Rac1 activation.
**TMEM59** (Transmembrane Protein 59)	Protein that potentiates the formation of the Wnt signalosome interacting with Fz and LRP.
**TORC1** (mTOR complex 1)	Complex involved in the regulation of protein synthesis. Wnt-induced GSK3 inhibition activates TORC1 and protein translation
**TSC2** **(TS complex subunit2)**	GTPase activating protein that modulates Rheb and TORC1 activity; Wnt-induced GSK3 inhibition blocks TSC2 phosphorylation and activates TORC1.
**Vav2**	Rac1 Guanosine exchange factor (GEF) involved in canonical Wnt-dependent Rac1 activation.
**Wnt** (Wingless-Type MMTV Integration Site Family)	Family of extracellular factors that bind to specific membrane receptor complexes to activate canonical or non-canonical signaling pathways
**YAP** **(Yes Associated Protein)**	Transcriptional factor negatively regulated by the Hippo pathway that promotes its nuclear export. Binds to β-catenin.
**Yes**	Protein kinase of the Src family. It phosphorylates YAP1 regulating the activity of the YAP1/TBX5/β-catenin complex.
